# PhyloPattern: regular expressions to identify complex patterns in phylogenetic trees

**DOI:** 10.1186/1471-2105-10-298

**Published:** 2009-09-19

**Authors:** Philippe Gouret, Julie D Thompson, Pierre Pontarotti

**Affiliations:** 1 UMR 6632, Evolutionary Biology and Modeling, University of Provence, 3 place Victor Hugo, 13331 Marseille, France; 2 IGBMC, (CNRS/INSERM/ULP), Biology and Structural Genomics Department, BP 10142, 67404 Illkirch Cedex, France

## Abstract

**Background:**

To effectively apply evolutionary concepts in genome-scale studies, large numbers of phylogenetic trees have to be automatically analysed, at a level approaching human expertise. Complex architectures must be recognized within the trees, so that associated information can be extracted.

**Results:**

Here, we present a new software library, PhyloPattern, for automating tree manipulations and analysis. PhyloPattern includes three main modules, which address essential tasks in high-throughput phylogenetic tree analysis: node annotation, pattern matching, and tree comparison. PhyloPattern thus allows the programmer to focus on: i) the use of predefined or user defined annotation functions to perform immediate or deferred evaluation of node properties, ii) the search for user-defined patterns in large phylogenetic trees, iii) the pairwise comparison of trees by dynamically generating patterns from one tree and applying them to the other.

**Conclusion:**

PhyloPattern greatly simplifies and accelerates the work of the computer scientist in the evolutionary biology field. The library has been used to automatically identify phylogenetic evidence for domain shuffling or gene loss events in the evolutionary histories of protein sequences. However any workflow that relies on phylogenetic tree analysis, could be automated with PhyloPattern.

## Background

Evolutionary concepts have revolutionized our understanding of biology [1]. Understanding evolution and especially genome evolution requires a global comparative approach in which individual genetic events are considered and integrated in their evolutionary context, which in turn may be correlated to the population history, the environment and the different phenomes. Phylogeny-based analysis provides an ideal framework for performing such investigations, by pinpointing when a genetic event occurred and by identifying the simultaneous occurrence of several events. Such correlations allow to highlight convergence and co-convergence (i.e. the apparition of the same events independently in biological history). Co-convergence can strengthen the cause-consequence effects between for example, two events and identify common selective pressures acting on the two events, as well as the functional or adaptive relationship between the corresponding characters [2,3]. In order to identify micro-evolutionary (sequence evolution) and macro-evolutionary (including gene duplication, deletion, exon shuffling, horizontal gene transfer) events, a detailed analysis of a phylogenetic tree is essential. Of course this can be performed by human experts, but such a manual analysis is impossible in the context of a large scale analysis project. Some state-of-the-art pipeline processes, such as the Figenix platform [4], the SIFTER platform [5] or the pipeline used to build the PhyloFacts database [6], are capable of producing phylogenetic trees on a large scale, so it should be possible to develop a new kind of automatic process using the phylogenetic trees as a starting point, in order to answer the biological questions mentioned above, using an evolutionary approach.

One important aspect of such automatic processes is an efficient traversal of the tree to identify genetic events for subsequent analysis. Hierarchical tree traversal is a common issue in computer science. In the biological field, phylogenetic trees are mostly traversed by hard coded algorithms and some programs, such as Rio Forester [7] allow the detection of duplication nodes and orthologs from a tree. Other tools allow the detection of specific genetic events: [8-13].

All of these tools include mathematical components which apply a probabilistic model to interpret the tree, and have led to some significant results. Unfortunately, current mathematical approaches cannot provide and integrate all human interpretations about trees, simply because some models have not been defined yet. One can cite, for example, the detection and localization of domain shuffling events in domain phylogenetic trees, which we will illustrate in this paper. As a consequence, it remains crucial for a biologist to apply his own knowledge and reasoning to the interpretation of phylogenetic trees. Clearly a software API is now needed, that can be easily integrated in automatic genome-scale processes to read phylogenetic trees, that provides a level of expertise as close as possible to that of the biologist, and that will facilitate the application of evolutionary approaches. Famfetch [14] is one such software that offers a simple pattern-matching system and includes a graphical user interface, allowing the user to define specific patterns.

However a biologist, reading a tree, is capable of recognising much more complex patterns. Indeed, the human expert, consciously or unconsciously, combines sophisticated phylogenetic architectures with constraints associated with the nodes' explicit or implicit properties. We call "explicit properties", properties already attached to the nodes when he reads it and we call "implicit properties", additional properties he must compute to complete the node's annotation.

The bioinformatician or the computer scientist working with the biologist would like to easily annotate the many phylogenetic trees he produces, and to define complex patterns for searching, without having to re-program the tree traversal each time.

Moreover "implicit properties" cannot always be evaluated immediately in a simple tree traversal. For example, to evaluate, for each node of a tree, a property such as the topological distance to a specific node of this tree, one needs to set an equation system between the unknown values of the property at each node during the tree traversal and then to solve it to get all values.

Finally, it should be possible to dynamically define the patterns from one tree, for example to compare tree structures.

Here, we describe a new compact software library, called PhyloPattern, which offers three main functional modules that can be used either independently or in combination:

- a tree annotation module, based on predefined traversal algorithms and predefined or user defined annotation functions, to produce immediate or deferred evaluations of node properties,

- a pattern matching module to define powerful patterns and to search for them in phylogenetic trees,

- a tree comparison module to globally compare rooted tree topologies or to identify matching nodes. These functionalities are illustrated in the present paper with biological examples.

## Implementation

### Representation of trees and patterns

Phylogenetic trees are composed of nodes linked in a hierarchical structure. Each terminal node, named a leaf, represents the biological object whose evolutionary history one wants to study. Each internal node then represents a real or virtual ancestor of all the leaves present in the node's subtree. Two nodes are connected by a branch, whose length gives complementary information about the predicted evolutionary distance between the nodes. In general, most phylogenetic trees are considered to be binary structures, where internal nodes own exactly two children. However, technically, phylogenetic trees are in fact n-ary structures, and tree construction algorithms can generate more than two children for an internal node if the true phylogenetic relationships cannot be determined. Such internal nodes are named rakes. Note that a rake can always be transformed into a binary structure by generating pseudo-nodes, although this may be problematic, since the pseudo-nodes cannot be interpreted and used directly.

PhyloPattern uses its own formalism to represent phylogenetic trees and patterns (the pattern formalism extends the tree formalism (see Table 1)), based on the syntax of a classic programming and Artificial Intelligence (AI) language: Prolog [15], which is particularly suitable for hierarchical structure manipulations. This important feature will be discussed in detail below.

**Table 1:** PhyloPattern syntax for representing trees and patterns

*<node>*	*::=*	*<leaf> | <internal_node> | <rake>*
*<leaf>*	*::=*	*" [" <empty_list>, <tag_list> "]"*

*<internal_node>*	*::=*	*" [" "[" <node>, <node> "]", <tag_list> "]"*

		

*<rake>*	*::=*	*&(" [" <node_list> "]")*

*<node_list>*	*::=*	*<node>*

	*::=*	*<node>, <node_list>*

		

*<empty_list>*	*::=*	*"[]"*

		

*<tag_list>*	*::=*	*<empty_list>*

	*::=*	*" [" <filled_tag_list> "]"*

		

*<filled_tag_list>*	*::=*	*<tag>*

	*::=*	*<tag>, <filled_tag_list>*

		

*<tag>*	*::=*	*<identifier> (<value>)*

		

*<leaf_list>*	*::=*	*<leaf>*

	*::=*	*<leaf>, <leaf_list>*

		

*<identifier>*	*::=*	*<lower-case-letter> { <letter> | <digit>}*

	*::=*	*' { <character> } '*

		

*<value>*	*::=*	*<identifier> | <number>*

		

*<pattern>*	*::=*	*<leaf>*

	*::=*	*" [" "[" <pattern>, <pattern> "]", <tag_list> "]"*

	*::=*	*&(" [" <pattern_list> "]")*

	*::=*	*@ (<pattern>)*

	*::=*	*$ (<pattern>)*

	*::=*	*#(" [" <leaf_list> "]")*

		

*<pattern_list>*	*::=*	*<pattern>*

	*::=*	*<pattern>, <pattern_list>*

The grammar rules used for describing trees and patterns are indicated using the BNF (Bacchus Naur Form: ) notation.

In addition to this formalism, PhyloPattern can read and write trees in NH  or NHX syntax (New Hampshire eXtended) [7].

For the PhyloPattern user, the formalism also provides "regular expression like" definitions for phylogenetic trees. Regular expressions are classically used to parse and find specified syntactic shapes inside sentences, without having to develop a specific syntactic analyser from user defined grammar rules. Typical examples are the regular expressions specified in Unix command shells .

In PhyloPattern, regular expressions are patterns used to find nodes with a specified architecture or according to specified criteria.

Note that the current version of PhyloPattern works only with rooted binary trees but accepts rake structures.

The syntax used in PhyloPattern for nodes in a phylogenetic tree is very simple. A node is expressed by:

[List_of_child_nodes, List_of_tags]

As in the NHX format, a "tag" refers to a property-name/property-value pair. Using this syntax, a leaf node is represented by:

[[], [tag1(Value1), tag2(Value2), ..., tagN(ValueN)]]

and an internal node is represented by:

[LeftChildNode, RightChildNode], [tag1(Value1), tag2(Value2), ..., tagN(ValueN)]]

where *LeftChildNode *and *RightChildNode *indicate the two child branches of the node. They are themselves nodes and so respect the same syntax. The denominations left and right have no biological significance and they are used for convenience only. Obviously and if it is necessary, left and right nodes are automatically permuted by PhyloPattern during matching phases.

We introduce another kind of syntax for rake nodes, which are non binary nodes with a zero length on each child branch:

&(List_of_nodes) <=> &([Node1, Node2, ..., NodeN])

A full BNF grammar of our formalism is provided in Table 1.

Constraints can also be associated with a pattern's structure. The basic syntax of a constraint is: *freeze (Variable, Predicate)*, but a more detailed explanation of how they work will be given below. Note that constraints, expressed separately from a pattern's structure, are not included in the grammar in Table 1.

### Prolog engine

As we said before, trees and patterns in PhyloPattern are in fact Prolog [15] language terms which allows us to implement all our API tools in this language.

Prolog belongs to the Artificial Intelligence (AI) language family. AI languages provide a means of modelling the knowledge and reasoning of humans, or any other intelligent species. Other examples of AI languages are Lisp [16], Camel [17], etc. PhyloPattern is not built with an algorithmic approach but rather on a first order logic approach which is the foundation of Prolog. All sentences in this language are expressed as logical implication rules between a set of facts which imply a single fact. The Prolog engine applies these rules in a backward chaining mode, which means that to verify that a fact is true, the engine searches for the facts that imply the given fact, and so on. The engine relies on two fundamental concepts namely: backtracking and unification. Backtracking is a mechanism which allows the generation of all solutions to a given question, i.e. to an initial fact that one wants to verify. This is sometimes called a Prolog clock, because the engine moves back and forth between future and past: the future when the engine tries to advance on a solution pathway and the past when it moves back on the path. This mechanism is very convenient for implementing solutions to problems where we need to explore a set of paths, some of these paths being dead-ends. Backtracking is well adapted to our pattern matching approach because matching cases are defined as crossroads that PhyloPattern encounters again and again, each time a tree's traversal reaches a new node. The unification concept is the fusion of a variable's assignation and equality concepts. Two Prolog terms are said to be "unifiable" if and only if they are equal or if all non assigned parts of one term can be assigned to an assigned part of the other term, thus implying the equality of fully assigned terms.

Here is a very small and trivial example that nevertheless gives a very complete illustration of all Prolog concepts applied to tree manipulations.

These four lines are extracted directly from the PhyloPattern source code:

% enumerate all subtrees from a tree

*subtree(Tree, Tree)*.

*subtree([LeftChild, _], _], Subtree) :- subtree(LeftChild, Subtree)*.

*subtree([_, RightChild], _], Subtree) :- subtree(RightChild, Subtree)*.

*subtree(&(List), Subtree) :- element(Tree, List), subtree(Tree, Subtree)*.

As specified in the comment line, this predicate is able to enumerate all subtrees existing in a tree. The aim here is not to give a complete explanation of the Prolog language but only a synthetic description of each rule. Thus, we consider each line in order: a tree is a subtree of a tree if it is the full tree, or a tree is a subtree of a tree if it is a subtree of the left child node, or a tree is a subtree of a tree if it is a subtree of the right child node, or a tree is a subtree of a tree if it is one of the elements of a rake.

PhyloPattern is a Prolog library, which can be used with any "Edimburg syntax like" [15] Prolog engine. The PhyloPattern toolbox can also be accessed from the Java language directly via the 'GNU Prolog for Java' API .

In addition, the 'GNU Prolog for Java' allows the definition of predicates directly in the Java language. Thus, the Rio Forester [7] parser is used to convert NHX trees to PhyloPattern Prolog terms.

A short example to show how a Prolog PhyloPattern script can be used from Java is given in the PhyloPattern package [see Additional file 1]. Other simple examples are provided with the PhyloPattern distribution (file src/samples.pl) to demonstrate how to run PhyloPattern and how to write scripts.

## Results

The strategy we adopted in the development of PhyloPattern, was first to understand how a biologist reads and uses a phylogenetic tree and, from this, we deduced three main functionalities that are essential for most tree analyses: tree annotation, pattern matching and trees comparison.

### Tree annotation module

An important functionality in PhyloPattern is the ability to assign crucial information to the nodes, before proceeding to the subsequent steps in the analysis. Indeed when a biologist reads a phylogenetic tree, he often incorporates complementary information, such as the species associated with each leaf, the fact that an internal node represents a speciation or a duplication event, or the domain architectures if leaves are associated with proteins.

More complex annotations can also be envisaged, for example, one would like to attribute to internal nodes: the list of all species present in the node's subtree or the distance to a specific node. The tree annotation module involves two distinct problems: the traversal of the tree in order to visit all nodes and the function used to annotate each node.

For the first problem, PhyloPattern offers two of the main traversal algorithms for binary trees: pre-order traversal and post-order traversal. The third major algorithm, namely in-order traversal, is *a priori *not useful for phylogenetic issues, but could be easily implemented. Pre-order traversal means "do what you have to do on the node itself, then on the left branch, and finally on the right branch". In contrast, postorder traversal means: "do what you have to do on the left branch first, on the right branch second and finally on the node itself". In-order traversal means "do what you have to do on the left branch, then on the node itself, and finally on the right branch". For example, for a very simple binary tree ((a, b)ab, c)abc, if the operation one wants to apply to each node is: "display its name", the different kinds of traversal will give the following results: for pre-order: *abc, ab, a, b, c*, for in-order: *a, ab, b, abc, c*, for post-order: *a, b, ab, c, abc*. The post-order approach performs a task first on the children, then on the parent node, which is logically the most useful kind of traversal for phylogenetic annotation, because usually we want to gather information from the leaves in order to propagate them to the internal nodes, i.e. we look at the present with the aim of understanding the past.

For the second problem, two kinds of annotation functions are distinguished: immediate evaluation functions and deferred evaluation functions. Immediate evaluation functions compute the value of a new tag for a node, when the traversal reaches it, for example, to assign to the node, in a post-order traversal, a list of all species present in its full subtree. By first performing the task on the node's children, it is easy to accumulate the species from the children to form the node's list of species.

**Figure 1:**
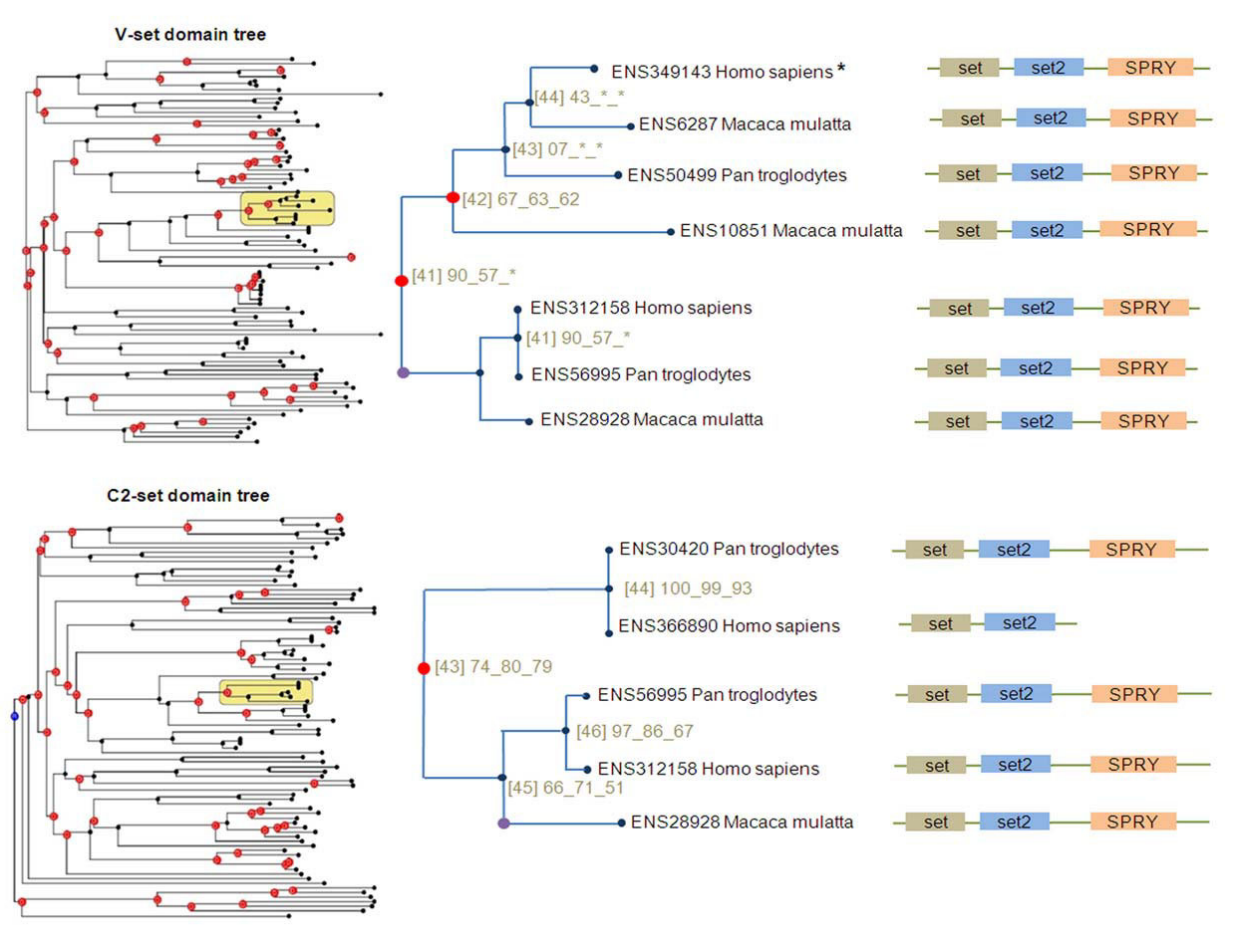
**Wired phylogenetic trees of V_set and C2_set domains (definitions from Pfam database **[21]**), respectively zoomed on nodes *41 *and *43 *(yellow areas)**. All protein sequences are from the Ensembl database [22]. The multiple alignment was built and annotated using the MACSIMS program [23] and the phylogeny was produced and visualized using the Figenix platform [4]. *[Number] *represents the identifier of the closest node. *Number1_Number2_Number3 *represents bootstrap values for the closest internal node (from three construction algorithms [4]). Red circles on nodes represent duplication events. Purple circles represent the detected homolog domains shuffling events. Labeled trees for V_set and C2_set domains are provided [see Additional file 2], [see Additional file 3].

Deferred evaluation is more complex, but can be used when the annotation function is unable to determine the value for a specific tag when the traversal reaches the node, although the function is able to install a constrained relation between the tag value for the node and the tag values for its children. For example, suppose that an evolutionary event has been detected on a branch of a tree (this kind of detection is described below, see yellow areas in Figure 1 (see nodes with asterisks) and suppose one wants to compare, at the DNA level, a sequence associated with this subtree and one associated with the other subtree of the event's parent node (the one which did not suffer the event).

It seems logical to choose the two sequences closest to the parent node, because they are normally less derived and should have fewer differences at the nucleotide level. To select these sequences in the tree, each node of the tree must be annotated with its total branch length to the event's parent node. When the post-order traversal reaches a node, it is impossible to set the distance value, but the annotation function can specify equations between the distance for the node and the distances for the children of this node: Call *d *the distance for a node, *dleft *and *dright*, distances for its children, *lleft *the length of the left branch and *lright *the length of the right branch. The annotation function can specify the two following equations: *d = dleft +/- lleft *and *d = dright +/- lright (/ means here the logical or)*.

When the function is called on the specific node against which one computes the distances, *d = 0*.

At the end of the post-order traversal however, a large set of equations has been defined and is automatically solved by PhyloPattern, associating a tag value with each node that is equal to the distance to the specified node. The use of this deferred evaluation mechanism means that many other types of equation or constraint can be applied, by defining an appropriate annotation function. In fact, any constraint expressible in the Prolog language could be implemented.

Note that the two annotation functions, given here as examples, are included in the PhyloPattern implementation. The PhyloPattern user is thus able to choose a predefined tree traversal mode (pre-order traversal or post-order traversal) and a predefined annotation function (immediate evaluation or deferred evaluation). He also has the possibility to define his own annotation function.

### Pattern matching module

Pattern recognition is the main task performed by a biologist when reading a phylogenetic tree. He is not always able to express explicitly and simply the patterns he uses, but if he succeeds, the patterns, in most cases, can be modelled and used within PhyloPattern. As mentioned before, in the PhyloPattern framework, a pattern's syntax is expressed in the same Prolog formalism as a phylogenetic tree, so any tree can itself be used as a pattern to search another tree. Subtrees are thus considered by PhyloPattern as sub-patterns. The PhyloPattern matching module takes as input: a tree term, a pattern term, and a list of constraints.

**Figure 2:**
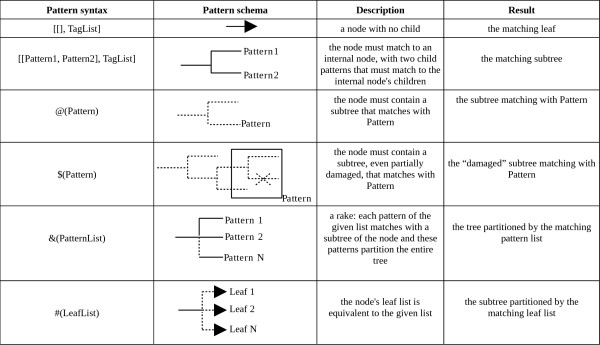
**Semantics of the PhyloPattern formalism**. Dotted lines indicate any tree containing the node, continuous lines indicate a specific existing branch in the tree.

To illustrate this, we can consider some basic sample patterns (without constraint), based on the forms given in Figure 2 and applied to the tree on the top right of Figure 1. Variables in patterns can be used to obtain results and the pattern matching function also returns a tree associated with the pattern. Note that _ is used as a mute variable in Prolog, i.e. a variable part whose value has no interest:

Using the following pattern:

@([[], [species('Macaca Mulatta'), name(X)]])

we obtain the solutions: *X = 'ENS6287' *or *X = 'ENS10851' *or *X = 'ENS28928'*,

and the results of the pattern matching function are exactly those leaves corresponding to the species "Macaca Mulatta".

Another example of a simple pattern, which can be used to search for the parent of a given node, is:

@([_, [_, [id('43')]]], [id(X), bootstraps(Y)]])

whose solutions are: *X = '42' *and *Y = '67_63_62'*,

and the result of the pattern matching function is the subtree corresponding to node 42.

The syntax of these patterns is discussed in detail below and a full list of patterns is given in Figure 2.

Depending on its location in the pattern, each variable part of the pattern can match with any subtree of the tree, with any list of tags or with any tag's value. Thus, the PhyloPattern matching module can be used either to find a match or to extract data from a tree. Note that tags expressed in the pattern must be included in the matching node's tags list, but that equality of tags list is not necessary.

Constraints associated with a pattern's structure must be verified before the match is accepted. A constraint is expressed as a Prolog free variable/Prolog predicate pair. The constraint is verified when the predicate is verified, and this verification occurs when the free variable is assigned. The output of the module includes the results of the pattern matching (see Figure 2), as well as the values of the free (without value) Prolog variables expressed in the pattern. A very important feature of PhyloPattern is that it always gives all solutions for a pattern applied to a tree.

A number of specific definitions have been introduced for patterns that increase their conciseness and their searching power. These definitions are listed in Figure 2 and are described in detail below.

The @(Pattern) syntax is used to find a specific sub-structure in a tree. For example, suppose one wants to search for orthologs of a specific sequence associated with a leaf of a tree. One must search for a speciation node with two child trees that respectively contain the given sequence and other sequences from a different species. This search can be performed with the following:

pattern structure:

@([@([[], OrthologTags]), @([[], SequenceTags])], ParentTags])

pattern constraints:

freeze([SequenceTags, element(name('sequence name'), SequenceTags)]), freeze([OrthologTags, element(species(OrthologSpeciesName), OrthologTags)]), freeze([SequenceTags, diff(OrthologSpeciesName, 'sequence species name')]), freeze([ParentTags, element(duplication(false), ParentTags)])

The structure definition is used to obtain all possible leaf pairs in the tree, which technically means "a node with a leaf coming from one of its children, and another leaf coming from the other child". The constraints can be expressed in natural language as: "*SequenceTags *contains a tag that is the name of the studied sequence", "*OrthologTags *contains a tag that is a species different from the one in *SequenceTags*", "*ParentTags *contains a non duplication tag".

As mentioned before, PhyloPattern provides all solutions matching the pattern, so if one applies this pattern to the tree associated to the V_set domain [see Additional file 2], for the studied sequence:

['lcl|ENSP00000383836|9606|HOMO~SAPIENS|', ...]

one obtains the ortholog leaves with tags:

*[name(lcl|ENSMUSP00000102170|10090|MUS~MUSCULUS|), id(183), length(0.05321),...]*,

*[name(lcl|ENSMUSP00000042662|10090|MUS~MUSCULUS|), id(184), length(0.05947),...]*,

*[name(lcl|ENSRNOP00000046201|10116|RATTUS~NORVEGICUS|), id(185), length(0.08274),...]*,

[name(lcl|ENSMUSP00000102174|10090|MUS~MUSCULUS|), id(186), length(0.11414),...],

[name(lcl|ENSRNOP00000043233|10116|RATTUS~NORVEGICUS|), id(187), length(0.1197),...]

The *#(LeafList) *syntax can be used to obtain all leaves for a tree, or to verify that a tree has a specified list of leaves.

The *$(Pattern) *syntax has a more complex interpretation. It can be used to find a specific subtree in a tree, even if the tree has to be "damaged" first, i.e. by eliminating some of its subtrees. This pattern can be used to create a new tree that consists of a subset of nodes of a given tree, where the subset is not a direct existing subtree of the tree. For example, suppose one has two trees associated with two protein domains and suppose one wants to study domain shuffling events based on these trees. To do this, one has to compare the tree topologies only for the sequences that are present in both trees, in order to detect differences in the evolutionary histories of the two domains. To obtain these trees with PhyloPattern, first a *#(LeafList) *pattern must be applied to each tree to obtain their respective leaf lists. Then the pattern: *$(#(CommonLeafList))*, where *CommonLeafList *is the intersection of the two leaf lists, must be applied to the two trees. To understand the *$(#(CommonLeafList)) *pattern, just cut it in two parts, the *$(_) *part enumerates all damaged trees in the given tree, the *#(CommonLeafList) *part ensures that the resulting trees contain exactly the list of leaves shared by the two trees.

Finally, the *&(PatternList) *term, used as a pattern (it is also a node syntax) allows to define a list of patterns to match a tree and to partition it. Usually this pattern is not used directly, but rather when one wants to globally compare two trees. (see the global tree comparison section below).

A simple example is provided here to demonstrate the flexibility of PhyloPattern, by chaining annotation phases and pattern matching phases with constraint definitions based on tags produced by annotations. Suppose one wants to identify shuffling events between two non homologous protein domains. An evolutionary strategy might be to construct phylogenetic trees for the two protein domains (based on a multiple sequence alignment) and then to perform the following steps with the PhyloPattern API:

#### Step 1

*annotate the leaves of the trees with the domain architectures of associated proteins (using a protein domain database) and annotate internal nodes of the trees by inferring their domain architectures from the leaf domain architectures (using for example the Dollo parsimony algorithm *[18]* or Maximum Likelihood methods *[19]),

#### Step 2

**Figure 3:**
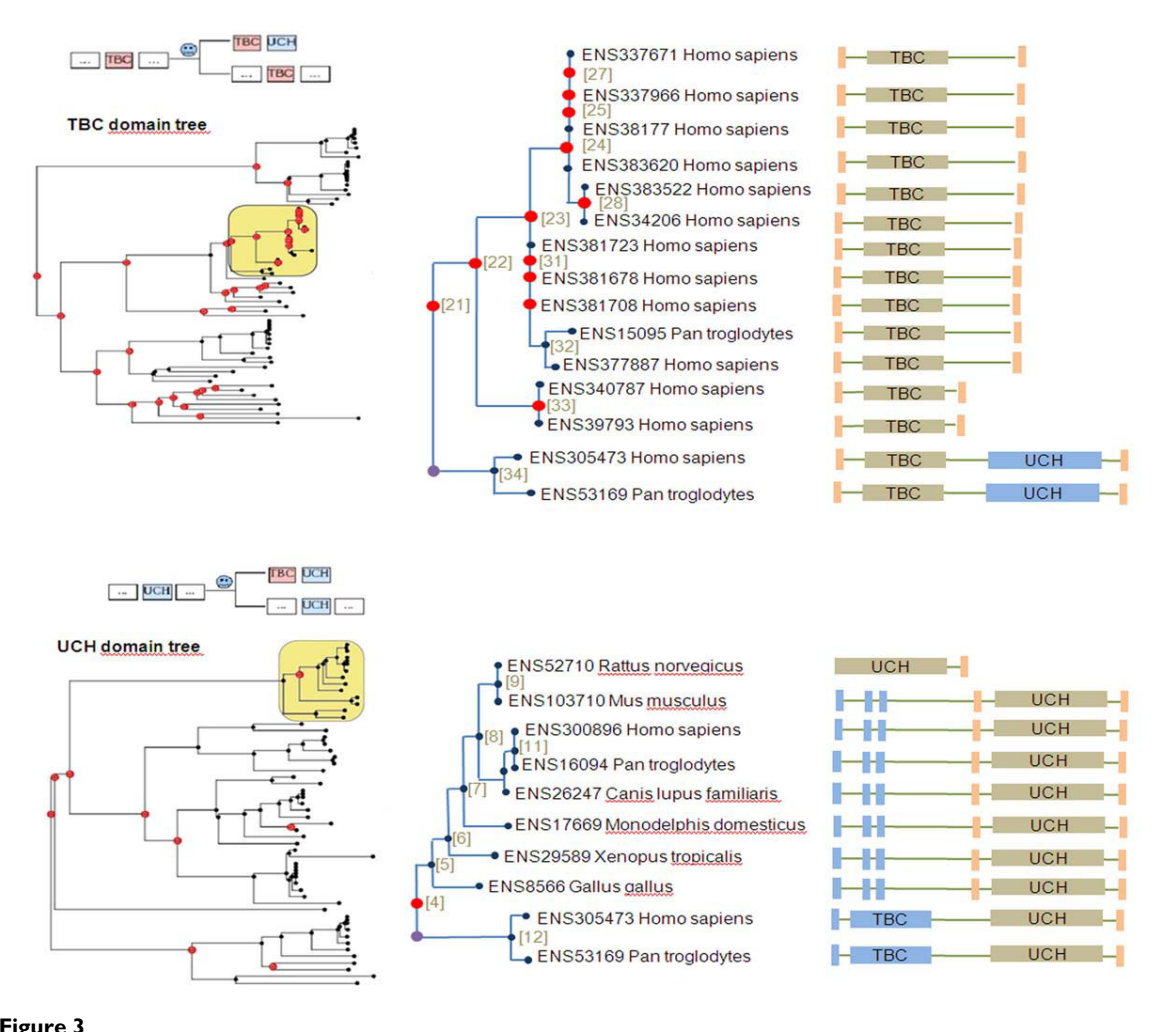
**Two phylogenetic trees are shown, which were built from the same protein sequence alignment, but that correspond to two different domains from the Pfam database **[21]: TBC and UCH. This example has already been described in the literature [24] and is used here as a benchmark for PhyloPattern. The results of each step in PhyloPattern (based on the strategy described in Pattern Matching) are shown. Step 1, Annotation: the full domain architecture is given for each sequence. Domain architectures for internal nodes are computed with the Dollo parsimony algorithm [18]. Step 2, Pattern Matching: the pattern shown above each tree is used to detect "parent" nodes of a shuffling event resulting in the architecture TBC-UCH (indicated in yellow). Step 3, Annotation: a purple circle is placed on the derived branch to locate the event. Step 4, Pattern Matching: A simple pattern is applied to extract leaves from each derived subtree. The human sequence ENSP305473 is common to the two subtrees and can be used as a reference for a subsequent genomic comparison with sequences having the "parent" architecture. Labeled trees for TBC and UCH domains are provided [see Additional file 4], [see Additional file 5].

*define a pattern with constraints mainly based on the domain architecture tag to try to find a parent node of a shuffling event and apply it to each tree (see pattern schema at the top of *Figure 3),

#### Step 3

*if such a node is found, annotate each tree, by adding event tags to derived nodes found under the event's parent node*,

#### Step 4

*apply two patterns to each tree; first to extract a common leaf (same name) from each "event marked" subtree and second to extract an "ancestral" leaf (with the "parent" domain architecture)*.

Potential shuffling events detected using this strategy could be verified by performing genomic comparisons of the common sequence with the two associated ancestral sequences. Figure 3 shows the results of this strategy for a domain shuffling event described in the literature.

### Trees comparison module

#### Global comparison

A "global comparison" can be used to determine whether two phylogenetic trees are equivalent. To do this with PhyloPattern, one of the trees is used as a pattern to be applied to the second tree. PhyloPattern includes a tool to transform a tree into a wired structure (nodes without tags except the node's name) in which badly supported nodes (nodes with "bad" bootstrap values, where thresholds are defined by the user) are collapsed to form rakes. This allows many alternative architectures to be matched with the "raked" pattern. As a result, when globally comparing two trees, it is possible to answer "yes" the two trees are "the same", even if the "badly supported" nodes do not have the same topology.

#### Local comparison

Another important tool in the PhyloPattern API, is the "local" tree comparison. In contrast to global tree comparisons, here the tool enumerates all the matching subtrees between the two trees. To do this, PhyloPattern dynamically defines patterns from all subtrees in one tree, "raking" some of them if necessary (see global comparison) and applies each to the second tree, using the pattern matching tool. One can imagine that these tasks can be very complex and would be very difficult and fastidious to reproduce by a human expert.

To continue the domain shuffling example introduced in the pattern matching section, when global matching fails on two independent domain trees, one can search for parent nodes of matching subtrees. By definition, these nodes have two children, one matching subtree and one non-matching subtree. Thus, one can infer that these nodes might be parents of a shuffling event, because the matching tree is associated with one set of sequences for one domain and to another set of sequences for the other domain. This implies two different evolutionary histories for two domains in an extant protein. On the trees shown in Figure 1, PhyloPattern has identified two such nodes with identifiers *41 *and *43*. Here, the subtrees with sequences: *[ENSP00000312158, ENSPTR00000056995, ENSMMUP00000028928] *match and the other subtrees do not.

## Conclusion

In this article, we have presented PhyloPattern, a software API, to:

(i) annotate easily phylogenetic trees using two predefined tree traversal algorithms and predefined or user defined annotation functions. Optional deferred evaluation of tags, in an annotation function, allows them to be computed by constraint system solving,

(ii) search and extract complex phylogenetic architectures and information using a "regular expression like" pattern syntax,

(iii) compare two trees, either globally using one as a pattern applied to the other, or locally by searching subtrees from one tree in the other. In this case, sub-patterns are dynamically and automatically defined by the tool.

PhyloPattern is designed to facilitate the in-depth analysis of a phylogenetic tree. To achieve this, we have tried to formalize and reproduce the studies currently performed by human experts. The goal is not simply to apply a mathematical algorithm, but to automate the complex evolutionary interpretation of phylogenetic trees for large-scale scientific pipelines. For example, it is currently being used in our laboratory in an automatic pipeline to detect genetic events occurring in vertebrate proteomes (manuscript in preparation). In this context, analyzing a tree with two hundred taxa takes only a few seconds. (test produced on a single Xeon 2,5 Ghz processor with 2Go of RAM). We also tried pattern matching on a relatively large tree (797 taxa) of the GPRC gene family which is one of the biggest in the TreePfam [20] database of phylogenetic trees. In this case, PhyloPattern took 150 seconds to search for all orthologs (see the orthology pattern in samples.pl file in the PhyloPattern package) of a given sequence in the full tree. Thus, we think that PhyloPattern represents a significant step towards automatic, high-throughput evolutionary biology studies.

Another potential application for automatic phylogenetic analysis would be in the case of very large trees (with thousands of taxa), where visual inspection would be impossible or too time consuming. Although PhyloPattern has not been designed specifically to handle such large trees, theoretically it should be possible on very powerful computers. Another possibility would be to divide a very large tree into a number of smaller subtrees (say 100 trees with 100 taxa) and to search for patterns recursively. For example, a strategy for an exhaustive domain shuffling events search, using PhyloPattern on a very large tree, could be defined like this:

1) Divide the large tree into sub trees with PhyloPattern, for example using a size criterion,

2) With PhyloPattern, search each sub tree individually to find nodes with new domain architectures due to a shuffling event,

3) Identify the domain structure of the ancestor of each subfamily,

4) Construct a new tree with one representative member for each subfamily,

5) With PhyloPattern, search this tree to find intermediate nodes with new domain architectures due to a shuffling event.

All the examples described in this paper illustrate the potential functionalities of PhyloPattern. Using the existing components in PhyloPattern in combination, we have shown that different genetic events can be localized on a phylogenetic tree, such as gene gain/loss, domain shuffling or gene transfers. With this information, two different complementary approaches can then be investigated: a gene centered approach or a global genetic approach. In the gene centered approach, a small number of trees are studied and the genetic events can be linked to different phenomes such as transcriptomes, interactomes, and phenotypes (note that a shift in a phenome can be also labeled on the tree using PhyloPattern). In a more global analysis, the genetic events can be robustly correlated with the phenotypic shift and in turn this can be linked to an environmental shift. Such an evolutionary based approach could also integrate multiple gene histories and possibly the whole genome. In this case, the deduced events and their functional consequences for one gene can be correlated with the other genes by comparing the tree annotations in order to find co-convergence between genetic events, function and environment.

In the future, PhyloPattern can be easily extended, by the addition of new predefined annotation functions and by the definition of new pattern syntaxes to solve specific issues not yet implemented in the current version.

## Availability and requirements

Project name: PhyloPattern

Project home page: 

Operating system(s): platform independent

Programming language: Prolog (Edimbourg) and Java (>= 1.5)

Other requirements: no

Licence: GPL

Any restrictions to use by non-academics: contact EBM Lab before using

## Abbreviations

API: Application Programming Interface; BNF: Bacchus Naur Form; NHX: New Hampshire eXtended

## Authors' contributions

PG specified PhyloPattern functionalities, developed the software and drafted the main parts of this manuscript. JDT provided alignment data to build trees and helped to validate PhyloPattern. PP highlighted the evolutionary concepts underlying the conception and drafted biological parts of this manuscript. All authors read and approved the final manuscript.

## Supplementary Material

Additional file 1 **PhyloPattern package, version 1.01**. The complete PhyloPattern package with runtime, sources, samples and documentation. Click here for file

Additional file 2 **V_set domain full phylogenetic tree**. The full domain tree corresponding to the top/left part of Figure 1. Click here for file

Additional file 3 **C2_set domain full phylogenetic tree**. The full domain tree corresponding to the bottom/left part of Figure 1. Click here for file

Additional file 4 **TBC domain full phylogenetic tree**. The full domain tree corresponding to the top/left part of Figure 3. Click here for file

Additional file 5 **UCH domain full phylogenetic tree**. The full domain tree corresponding to the bottom/left part of Figure 3. Click here for file

## References

[B1] Dobzhansky T (1973). Nothing in Biology Makes Sense Except in the Light of Evolution. The American Biology Teacher.

[B2] Levasseur A, Orlando L, Bailly X, Milinkovitch MC, Danchin EG, Pontarotti P (2007). Conceptual bases for quantifying the role of the environment on gene evolution: the participation of positive selection and neutral evolution. Biol Rev Camb Philos Soc.

[B3] Barker D, Pagel M (2005). Predicting functional gene links from phylogenetic-statistical analyses of whole genomes. PLoS Comput Biol.

[B4] Gouret P, Vitiello V, Balandraud N, Gilles A, Pontarotti P, Danchin EG (2005). FIGENIX: intelligent automation of genomic annotation: expertise integration in a new software platform. BMC Bioinformatics.

[B5] Engelhardt BE, Jordan MI, Muratore KE, Brenner SE (2005). Protein molecular function prediction by Bayesian phylogenomics. PLoS Comput Biol.

[B6] Krishnamurthy N, Brown DP, Kirshner D, Sjölander K (2006). PhyloFacts: an online structural phylogenomic encyclopedia for protein functional and structural classification. Genome Biol.

[B7] Zmasek CM, Eddy SR (2002). RIO: analyzing proteomes by automated phylogenomics using resampled inference of orthologs. BMC Bioinformatics.

[B8] Sakarya O, Kosik KS, Oakley TH (2008). Reconstructing ancestral genome content based on symmetrical best alignments and Dollo parsimony. Bioinformatics.

[B9] Durand D, Halldórsson BV, Vernot B (2006). A hybrid micro-macroevolutionary approach to gene tree reconstruction. J Comput Biol.

[B10] Beiko RG, Hamilton N (2006). Phylogenetic identification of lateral genetic transfer events. BMC Evol Biol.

[B11] Huson DH, Bryant D (2006). Application of phylogenetic networks in evolutionary studies. Mol Biol Evol.

[B12] Blomme T, Vandepoele K, De Bodt S, Simillion C, Maere S, Peer Y Van de (2006). The gain and loss of genes during 600 million years of vertebrate evolution. Genome Biol.

[B13] Arvestad L, Berglund AC, Lagergren J, Sennblad B (2003). Bayesian gene/species tree reconciliation and orthology analysis using MCMC. Bioinformatics.

[B14] Dufayard JF, Duret L, Penel S, Gouy M, Rechenmann F, Perrière G (2005). Tree pattern matching in phylogenetic trees: automatic search for orthologs or paralogs in homologous gene sequence databases. Bioinformatics.

[B15] Warren DHD, Pereira LM, Pereira F (1977). Prolog the language and its implementation compared with Lisp. Symposium on Artificial Intelligence and Programming Languages, Rochester, NY.

[B16] McCarthy J (1960). Recursive Functions of Symbolic Expressions and Their Computation by Machine, Part I.

[B17] Wright AK, Fellensein M (1992). A Syntactic Approach to Type Soundness. Information & Computation.

[B18] Farris JS (1977). Phylogenetic analysis under Dollo's law. Syst Zool.

[B19] Felsenstein J (1981). Evolutionary trees from DNA sequences: a maximum likelihood approach. J Mol Evol.

[B20] Ruan J, Li H, Chen Z, Coghlan A, Coin LJ, Guo Y, Hériché JK, Hu Y, Kristiansen K, Li R, Liu T, Moses A, Qin J, Vang S, Vilella AJ, Ureta-Vidal A, Bolund L, Wang J, Durbin R (2008). TreeFam: 2008 Update. Nucl Acids Res.

[B21] Bateman A, Birney E, Durbin R, Eddy SR, Howe KL, Sonnhammer EL (2000). The Pfam protein families database. Nucleic Acids Res.

[B22] Hubbard TJ, Aken BL, Ayling S, Ballester B, Beal K, Bragin E, Brent S, Chen Y, Clapham P, Clarke L, Coates G, Fairley S, Fitzgerald S, Fernandez-Banet J, Gordon L, Graf S, Haider S, Hammond M, Holland R, Howe K, Jenkinson A, Johnson N, Kahari A, Keefe D, Keenan S, Kinsella R, Kokocinski F, Kulesha E, Lawson D, Longden I, Megy K, Meidl P, Overduin B, Parker A, Pritchard B, Rios D, Schuster M, Slater G, Smedley D, Spooner W, Spudich G, Trevanion S, Vilella A, Vogel J, White S, Wilder S, Zadissa A, Birney E, Cunningham F, Curwen V, Durbin R, Fernandez-Suarez XM, Herrero J, Kasprzyk A, Proctor G, Smith J, Searle S, Flicek P (2009). Ensembl 2009. Nucl Acids Res.

[B23] Thompson JD, Muller A, Waterhouse A, Procter J, Barton GJ, Plewniak F, Poch O (2006). MACSIMS: multiple alignment of complete sequences information management system. BMC Bioinformatics.

[B24] Paulding CA, Ruvolo M, Haber DA (2003). The Tre2 (USP6) oncogene is a hominoid-specific gene. Proc Natl Acad Sci USA.

