# Supplementary figures and images for: PhyloPattern: regular expressions to identify complex patterns in phylogenetic trees
Source: BMC Bioinformatics. 2009 Sep 19;10:298. doi: 10.1186/1471-2105-10-298 (PMC2759962; doi:10.1186/1471-2105-10-298)

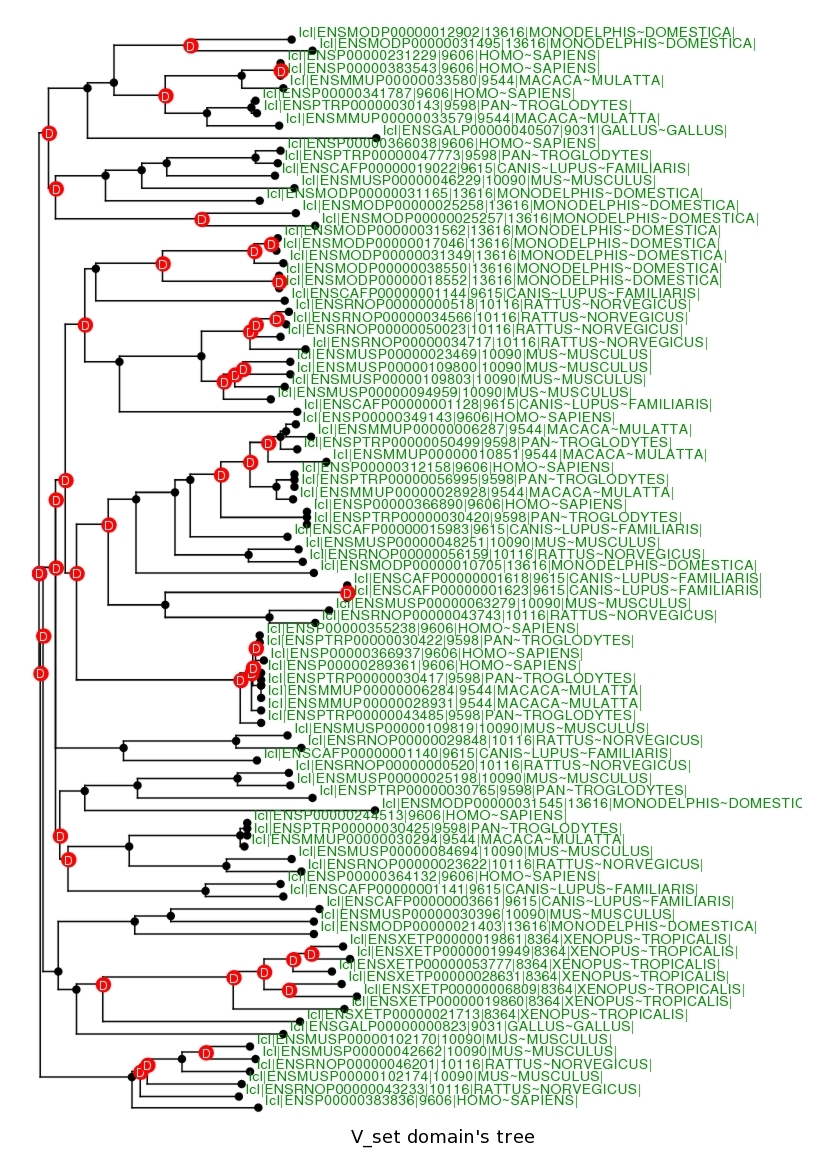

Supplement: Additional file 2 — V_set domain full phylogenetic tree. The full domain tree corresponding to the top/left part of Figure 1. [file 1471-2105-10-298-S2.JPEG]

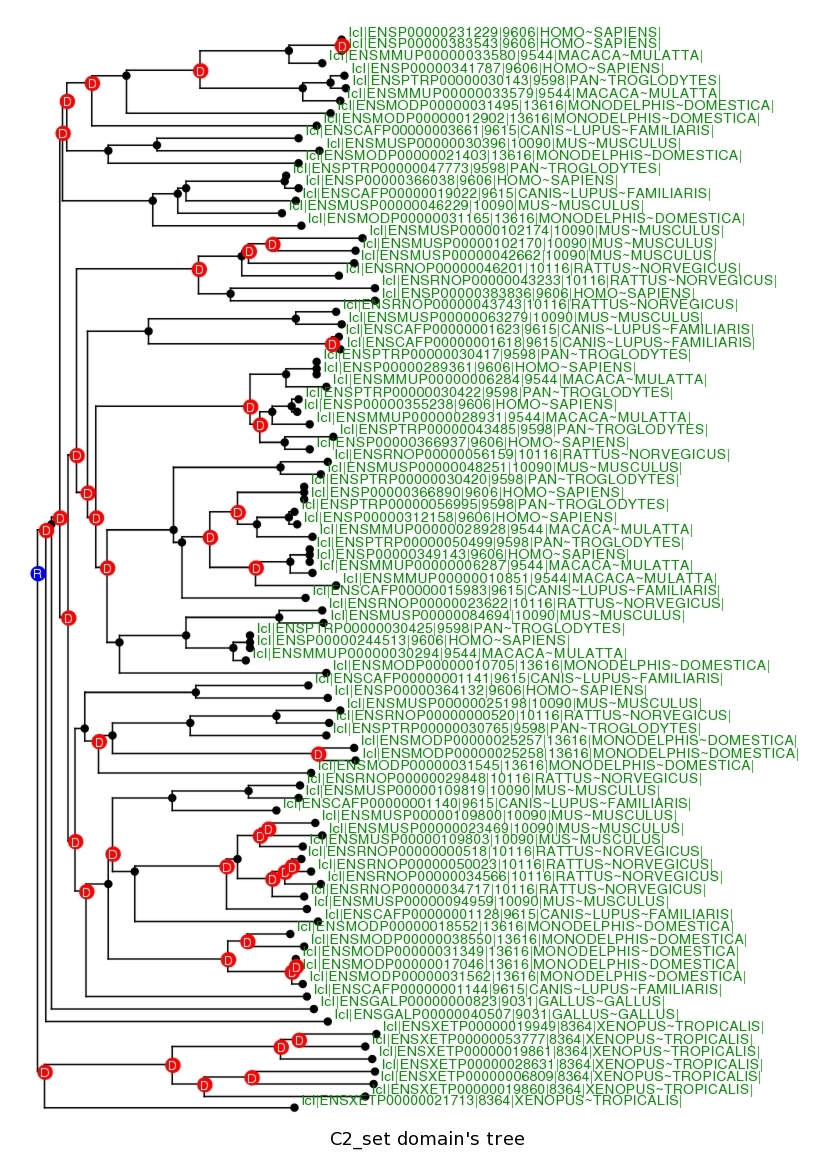

Supplement: Additional file 3 — C2_set domain full phylogenetic tree. The full domain tree corresponding to the bottom/left part of Figure 1. [file 1471-2105-10-298-S3.JPEG]

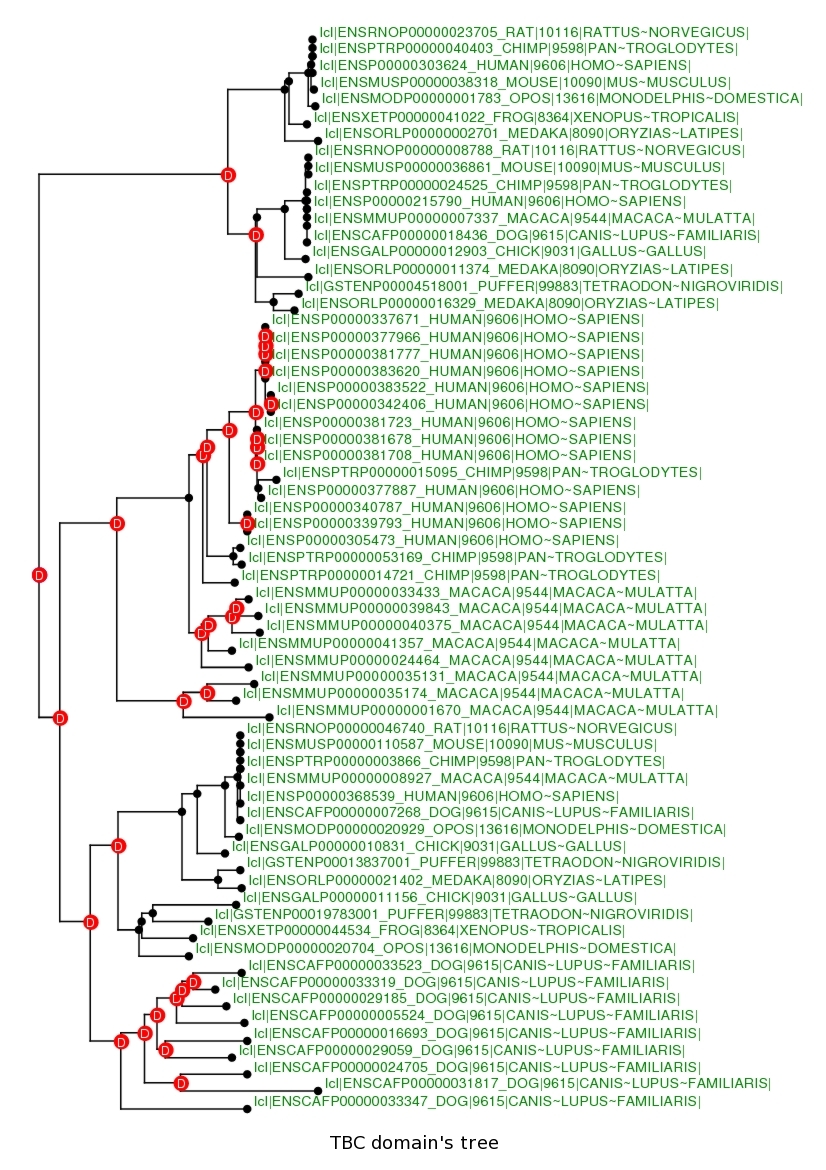

Supplement: Additional file 4 — TBC domain full phylogenetic tree. The full domain tree corresponding to the top/left part of Figure 3. [file 1471-2105-10-298-S4.jpeg]

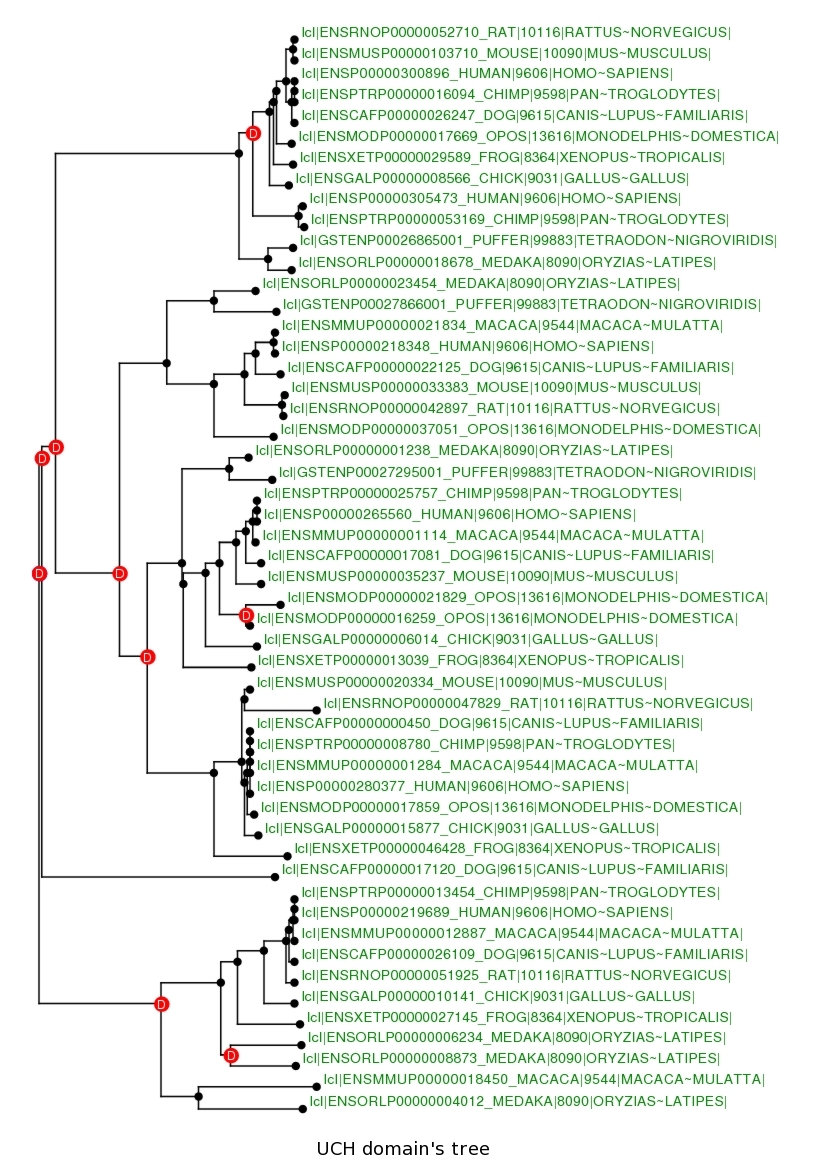

Supplement: Additional file 5 — UCH domain full phylogenetic tree. The full domain tree corresponding to the bottom/left part of Figure 3. [file 1471-2105-10-298-S5.jpeg]
